# A scoping review of diagnostic techniques used for the detection of peri-implantitis around endosseous dental implants

**DOI:** 10.3389/fdmed.2025.1722375

**Published:** 2026-01-09

**Authors:** Arvind Ramanathan, Shobha J. Rodrigues, Sandipan Mukherjee, Ramya Kudpi Shenoy, Shushma B. Rao, Pooja Rao, Umesh Y. Pai, Vidya Kamalaksh Shenoy

**Affiliations:** 1 Department of Oral & Maxillofacial Surgery, Manipal College of Dental Sciences Mangalore, Manipal Academy of Higher Education, Manipal, India; 2 Department of Prosthodontics & Crown & Bridge, Manipal College of Dental Sciences Mangalore, Manipal Academy of Higher Education, Manipal, India; 3 Department of Public Health Dentistry, Manipal College of Dental Sciences Mangalore, Manipal Academy of Higher Education, Manipal, India; 4 Department of Microbiology, Kasturba Medical College Mangalore, Manipal Academy of Higher Education, Manipal, India; 5 Department of Prosthodontics & Crown & Bridge, A J Institute Dental Sciences Mangalore, Karnataka, India

**Keywords:** endosseous implants, peri implant diagnosis, peri implant mucositis, periimplantitis, scoping review

## Abstract

**Introduction:**

Periimplant mucositis and periimplantitis are inflammatory reactions occurring around endosseous dental implants. The objective of this scoping review was to examine diagnostic techniques used to assess periimplantitis in clinical studies on human subjects.

**Methodology:**

The research question formulated was “What are the various diagnostic techniques used for detection of periimplantitis in patients who have received endosseous dental implants?” MEDLINE (PubMed), EMBASE, and SCOPUS were searched using a combined MeSH-based search strategy for studies published between 2015 to February 2025.

**Results:**

A total of 162 unique studies were included. Study designs, publication years, diagnostic domains, and methodological characteristics are summarized. Studies were categorized as belonging to the diagnostic domains of Imaging (*n* = 11), Microbial profiling (*n* = 57), Biomarkers in PICF/saliva (*n* = 28), Metabolic (*n* = 38), Genetic (*n* = 15) and Histopathology (*n* = 13). All studies used clinical and radiographic criteria to diagnose periimplantitis and then further assessed novel techniques and protocols for early diagnosis. Though standardized intraoral periapical (IOPA) radiograph remains the clinical standard of assessing peri implant bone loss, intraoral ultrasonography demonstrates potential utility to assess both peri implant hard and soft tissues. Microbial studies use Quantitative Polymerase Chain Reaction and Gene Sequencing techniques to identify bacterial community structures and microbial “shifts” that trigger inflammatory responses and measure therapeutic effects of treatments of established disease. Biomarkers of inflammation Interleukin-1β (IL-1β), collagen degradation enzymes active-Matrix Metalloproteinases-8 (aMMP-8) and bone turnover marker Receptor activator nuclear factor kappa b ligand (RANKL) in PICF demonstrate potential diagnostic and prognostic utility. Molecular signatures and study of small molecules were used for discovery of novel biomarkers while genetic studies assessed genetic polymorphisms increasing susceptibility to periimplantitis. Pathologic studies assessed changes in tissue architecture and correlation of shed implant particles and peri implantitis.

**Conclusions:**

This scoping review identified major diagnostic domains and mapped various diagnostic tools to provide an overview of contemporary diagnostic approaches.

## 1 Introduction

Endosseous dental implants are now a standard of care in the prosthodontic rehabilitation of edentulous and partially edentulous patients. The increasing availability of advanced technology and clinical expertise has established implant-based therapy as the preferred treatment for tooth loss (1). However, implants are susceptible to inflammatory complications. Bacterial adhesion on implant surfaces can trigger an inflammatory response that, if unaddressed, culminate in crestal bone loss and subsequent implant failure (2).

This pathology exists on a spectrum encompassing two distinct clinical entities. The first, peri-implant mucositis, is characterized by inflammation of the peri-implant soft tissues without associated bone loss. The second, peri-implantitis, involves soft tissue inflammation accompanied by clinical and radiographic evidence of bone deterioration. The formal diagnostic criteria for both conditions were definitively established by the 2017 World Workshop on the Classification of Periodontal and Peri-Implant Diseases and Conditions (3).

These clinico-radiographic findings represent the endpoint of a molecular cascade. This process involves the formation of signaling molecules that initiate or mediate the pathological mechanisms leading to clinical disease. The detection of these molecular intermediates offers a promising avenue for predicting disease onset before irreversible clinical damage occurs, thereby enabling early intervention to prevent implant failure. Consequently, current research is actively exploring novel diagnostic approaches, including methods to identify shifts in the peri-implant microbiome, pinpoint specific pathogenic microorganisms, and assess genetic polymorphisms that may predispose individuals to the development or progression of peri-implant diseases.

Given the diverse and rapidly evolving landscape of these diagnostic techniques, a comprehensive synthesis of the literature is required. To this end, we performed a scoping review to systematically map the progress in this field across various study designs. Our review was guided by the following research question: “What are the various diagnostic techniques used for the detection of peri-implant mucositis/peri-implantitis in human subjects who have received endosseous dental implants over the past decade?”

## 2 Methods

### 2.1 Protocol

A protocol was developed before initiation of the review, with feedback from all research team members and managed using the Rayyan.ai platform. This scoping review was designed and conducted following the Preferred Reporting Items for Systematic reviews and Meta-Analyses extension for Scoping Reviews (PRISMA-ScR) Checklist (4). The research question was “What are the Various Diagnostic Techniques used for the detection of Peri-implantitis”? The search was conducted following the PCC framework.

Population: Patients who had received endosseous dental implants.

Concept: various diagnostic techniques used for periimplantitis.

Context: All studies published in English language between 2015 and February 2025.

### 2.2 Eligibility criteria

Inclusion criteria

- Comparative studies involving human volunteers
- Clear diagnostic techniques for peri implant mucositis/periimplantitis
- Randomized controlled trials, Observational studies, Case control studies, Quazi experimental studies, case series, case reports
- Published in English language in the last 10 years

Exclusion criteria

- Diagnostic methods were not clearly defined
- Reviews
- studies published prior to the review period
- Animal studies, Invitro studies

### 2.3 Search strategy

**Figure 1:**
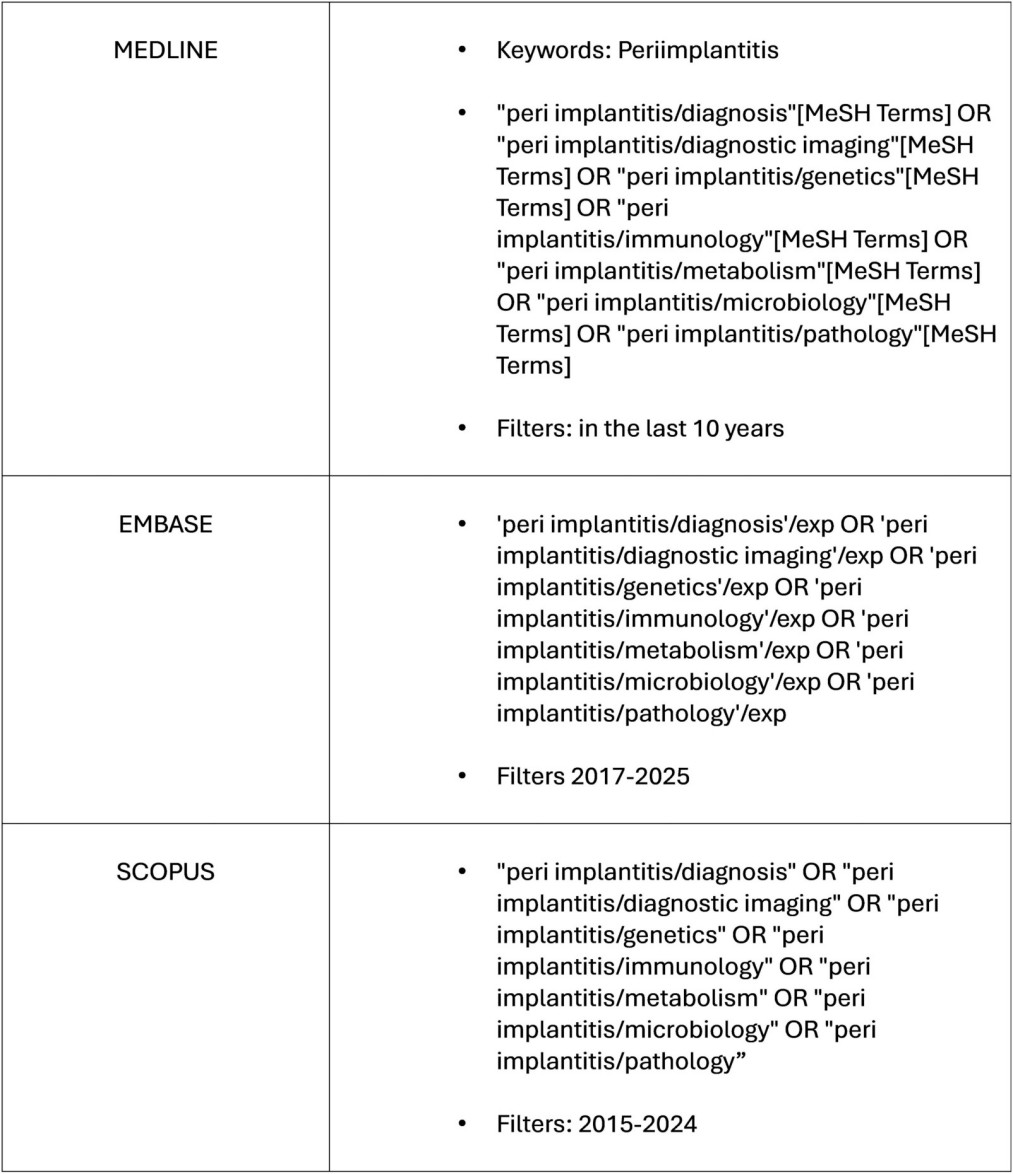
Summary of database search strategy.

MEDLINE (PubMed), EMBASE, and SCOPUS were searched on 12 February 2025 using a combined MeSH-based strategy adapted via the Polyglot Search Translator. Database-specific search strategies, including Boolean operators and applied filters, are provided in Figure 1. Searches were limited to English-language publications between 2015 and February 2025. This search string was then adapted for EMBASE and SCOPUS using the Polyglot Search Translator (Figure 1).

## 3 Study selection and data extraction

Four reviewers independently screened titles and abstracts. Two reviewers independently screened full texts. Disagreements were first resolved through discussion; unresolved discrepancies were adjudicated by a senior author. Reasons for exclusion at full-text stage were documented.

Data extraction was conducted using predefined forms describing study design, year, diagnostic method, measurement characteristics, and major findings. As recommended by PRISMA-ScR, no formal risk-of-bias assessment was conducted due to the mapping nature of this review. The findings from each critically appraised article are presented in tabular form and are accompanied by a narrative synthesis.

## 4 Results

**Figure 2:**
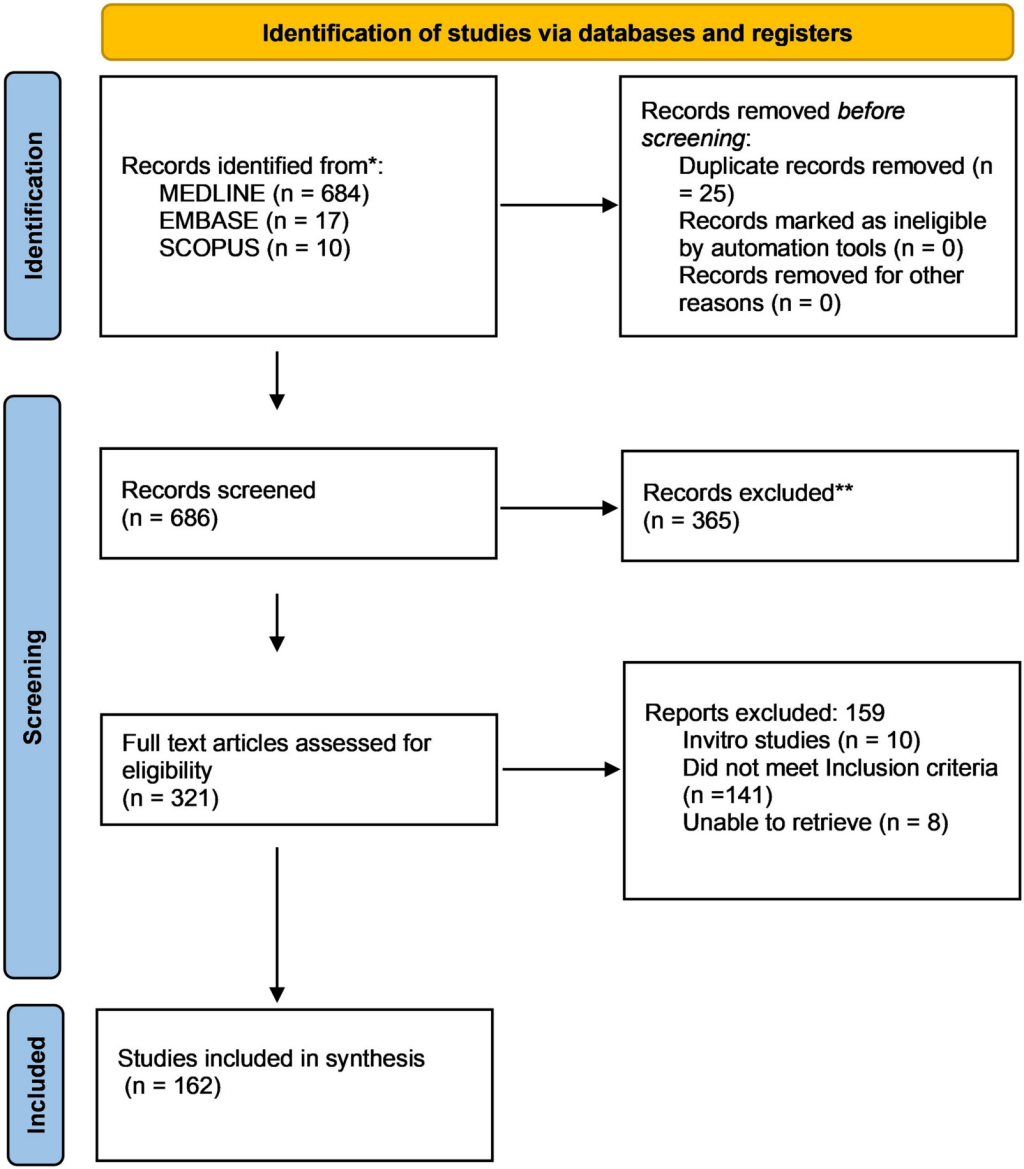
Flow chart of literature search.

The outcomes of the literature search and study selection process are summarized in a flow diagram adapted from the PRISMA guidelines (Figure 2).

We were able to categorize search results under the following 6 groups, with the type of studies in each group also mentioned (Table 1).

**Table 1:** Study categorizations.

Category	Number of studies	Type of studies	Table number
Diagnostic imaging of periimplantitis	11	Retrospective 4 Case control 2 Controlled clinical trial 1 Cross sectional 1 Case report 1 Case series 1 Pilot 1	Table 2
Microbiological diagnostics in periimplantitis & Microbial diagnostics after periimplantitis therapy	34 (&) 23	Cross sectional 13 Case control 13 Pilot 3 Randomized Controlled Trial 1 Cohort 1 Comparative 1 Preliminary study 1 Case series 1 Randomized controlled Trial 11 Cohort 3 Pilot 2 Retrospective 2 Cross sectional 1 Case control 1 Prospective 1 Observational 1 Comparative 1	Table 3 Table 4
Diagnostic biomarkers of periimplantitis in PICF/saliva	28	Comparative 8 Cross sectional 7 Case control 6 Randomized controlled trial 3 Prospective 3 Pilot 1	Table 5
Metabolic diagnostic techniques in periimplantitis	38	Case Control 10 Cross sectional 9 Pilot 7 Evaluation 6 Retrospective 2 Observational 2 Transversal 1 Discovery 1	Table 6
Genetics in periimplantitis diagnosis	15	Case control 10 Cross sectional 4 Pilot 1	Table 7
Pathologic diagnostic techniques for periimplantitis	13	Case control 5 Cross sectional 4 Cohort 1 Proof of concept 1	Table 8

Further appraisal of individual articles within each grouping was performed and the results are tabulated in the following tables in chronological sequence (Tables 2–8).

**Table 2:** Diagnostic imaging of periimplantitis.

Study	Imaging technique	Outcome
Galindo-Moreno et al. 2015 (5) Retrospective study	Intraoral Periapical (IOPA) Radiographs	Early bone loss in the healing and immediate post loading period negatively influenced outcome. Marginal bone loss of more than 0.4 mm per year was indicative of peri implant bone loss progression
Christiaens et al. 2017 (6) Controlled clinical trial	Intraoral Periapical (IOPA) Radiographs	Inferior quality of peri implant bone level assessment and under diagnosis. Bone sounding without flap elevation gave the most accurate assessments
Bender et al. 2017 (7) Retrospective study	Cone Beam Computed Tomography (CBCT)	May be used to assess the bone defect morphology of peri implantitis, but only after initial clinical examination and Two-dimensional imaging
Schwarz et al. 2017 (8) Cross sectional study	Ultrasonic biometer A scan	Significant increase of horizontal mucosal thickness at peri implantitis sites
Izzetti et al. 2019 (9) Case report	Ultra-High Frequency Ultrasonography (UHFUS)	To assess peri-implant soft tissues, which can reveal alterations in periosteum. UHFUS is a potential tool for the study of peri implantitis
Galarraga-Vinueza et al. 2020 (10) Pilot study	Intraoral scans	Describe the volumetric soft tissue changes that occurred in peri implantitis
Thöne-Mühling et al. 2021 (11) Case series	Intraoral High resolution Ultrasound probe	Real time imaging of crestal bone level and soft tissue volume around dental implants with peri implantitis
Barootchi et al. 2022 (12) Case control study	Ultrasound's quantified Color Velocity and Color Power	correlated directly with clinical assessments of implant health, peri implant mucositis and peri implantitis, thereby using dynamic tissue perfusion and blood flow variations to assess peri implantitis
Muraoka et al. 2023 (13) Retrospective study	Magnetic Resonance Imaging (MRI)	Peri-implantitis causes non-neoplastic lymphadenopathy
Galarraga-Vinueza et al. 2024 (14) Case control study	High Frequency Ultrasound (HFUS) echo intensity	Hypoechoic Supra-crestal Area (HSA) is a valid ultrasonographic diagnostic marker of peri implantitis
Pons et al. 2024 (15) Retrospective cohort study	Cone Beam Computed Tomography	Association between posterior maxillary implants afflicted with peri implantitis and antral membrane thickening

**Table 3:** Microbial diagnostics in periimplantitis.

Study	Sample	Microbial diagnostic techniques	Microorganisms in peri-implant health	Microorganisms in peri-implant mucositis	Microorganisms in Peri-implantitis
Tsigarida et al. 2015 (16) Case control study	Peri implant biofilms	454 -pyrosequencing	Smokers exhibit lower microbial diversity	Capnocytophaga, Lactobacillus, Leptotrichia, Prevotella	Capnocytophaga, Treponema, Propionibacterium, Pseudomonas, Lactobacillus and Leptotrichia. Lactobacilli, Propionibacteria, and Rothia exclusive to smokers
Neilands et al. 2015 (17) Case control study	Periimplant biofilm	Culture techniques			Porphyromonas, Prevotella and anaerobic gram-positive cocci
Zhuang et al. 2016 (18) Case control study	Sub gingival plaque	Quantitative Real Time - Polymerase Chain Reaction (PCR)			P. Gingivalis and f. Nucleatum were significantly associated with periodontitis, but not with peri-implantitis Aggregatibacter actinomycetemcomitans was associated with both
Canullo et al. 2016 (19) Cross sectional study	Plaque biofilm Inner implant connections	Quantitative Real Time PCR			Porphyromonas gingivalis (Pg), Tannerella forsythensis (Tf), Prevotella intermedia (Pi), Peptostreptococcus micros (Pm), Eikenella corrodens (Ec)
Yu et al. 2016 (20) Case control	Subgingival plaque sample	PCR	Synergistetis taxa	Synergistetis taxa	Synergistetis taxa
Schmalz et al. 2016 (21) Comparative study	Submucosal sample	PCR to Real Time PCR			No significant correlations between the bacterial and disease patterns, so the benefit of using microbiological tests for the diagnosis of peri-implant diseases is questionable
Ata-Ali et al. 2016 (22) Cross sectional	Peri implant sulcus	IAI - Pado Test 4.5 (IAI)			T forsythia, P gingivalis and T denticola and A actinomycetemcomitans
Apatzidou et al. 2017 (23) Case control study	Plaque	High throughput sequencing using Illumina Miseq platform	Greater diversity of microbial flora. Genera actinobacillus and streptococcus		Prevotella and porphyromonas Synergistetes
Sanz-Martin et al. 2017 (24) Case control study	Submucosal biofilms	16s rRNA gene sequencing Miseq Illumina sequencing	Genera actinobacteria, rothia and neisseria		Depleted of commensals and enriched by pathogens like bacteroides, spirochetes, synergistetes, porphyromonas, treponema, fusobacterium, parvimonas, campylobacter
Gürlek et al. 2017 (25) Cross sectional	Submucosal plaque	Real Time PCR	Actinomyces naeslundi, streptococcus oralis		Prevotella intermedia, treponema denticola, prevotella oralis
Ziebolz et al. 2017 (26) Cross sectional	Peri implant crevicular fluid	PCR			Treponema denticola and prevotella intermedia
Schincaglia et al. 2017 (27) Pilot study	Sub gingival microbiome	16s -rRNA gene sequencing	Rothia, Streptococcus and Actinomyces		Veillonella, Prevotella, Fusobacterium and Treponema
De Waal et al. 2017 (28) Case Control Study	Peri implant submucosa	Culturing techniques	While Aggregatibacter actinomycetamcomitans and Staphylococcus		Porphyromonas gingivalis, Prevotella intermedia, Tanerella forsythia and Fusobacterium nucleatum
Canullo et al. 2017 (29) Case Control Study	Inner part of implant connection, plaque samples from peri implant pockets	Real Time PCR	Lesser number of bacteria		Increased no. Of bacteria Parvimonas micra
Mencio et al. 2017 (30) Randomized Controlled Trial	Peri-implant microflora using paper points inserted in peri-implant sulcus for 30 s	Real Time PCR			Implants with screwed connection showed a higher risk of peri-implantitis than implants with cemented connection. Bacterial colonization of peri-implant sulci was over the pathogenic threshold for 5 bacteria
Tenenbaum et al. 2017 (31) Cohort study	Subgingival microbial flora	DNA-DNA checkerboard hybridization			Porphyromonas gingivalis, Prevotella intermedia, Actinomyces naeslundi, Eikenella corrodens Campylobacter rectus
Pimentel et al. 2018 (32) Case Control study	Submucosal plaque	Pyrosequencing	Actinomyces, Capnocytophaga and Streptococcus Smoking negatively affects peri-implant health		Fusobacterium, Tannerella and Mogibacterium
Daubert et al. 2018 (33) Cross sectional study	Submucosal plaque	16S rRNA gene sequencing	Streptococcus, Prevotella and Hemophilus		Veilonella
Eckert et al. 2018 (34) Pilot study	Submucosal plaque	Quantitative PCR			Porphyromonas gingivalis and Treponema forsythia and their proteases
Al-Ahmad et al. 2018 (35) Case control study	Subgingival plaque	16S rRNA gene cloning and PCR			Anaerobic gram negative periopathogens, Porphyromonas gingivalis and Treponema forsythia
Yeh et al. 2019 (36) Preliminary study	Peri implantitis pockets using paper points	Matrix Assisted Laser Desorption/Ionization - Time of Flight Mass Spectrometry (MALDI-TOFMS)			Neisseria flavescens, Streptococcus constellatus, Slackia exigua, Streptococcus intermedius, Fusobacterium nucleatum and Gemella morbillorum
Ghensi et al. 2020 (37) Case control study	Plaque microbiome	Strain resolution metagenomic sequencing		Fusobacterium nucleatum acting as a keystone colonizer.	Periimplantitis Related Complex (PIRC) composed of seven species namely Porphyromonas gingivalis, Tanerella forsythia, Treponema denticola, Porphyromonas endodontalis, Fusobacterium fastidiosum, Prevotella intermedia and Fusobacterium nucleatum
Zhou et al. 2022 (38) Case Control study	Peri implant plaque	Gene sequencing using Illumina HiSeq platform	Genera Actinomyces and Streptococcus	Fusobacterium and Prevotella potential microbial markers in peri implant mucositis	
Barbagallo et al. 2022 (39) Cross-sectional pilot study	plaque	16S rRNA gene sequencing	Lautropia, Rothia, Capnocytophaga and Kingella		Peptostreptococcaceae, Dialister, Mongibacterium, Atopobium and Filifactor
Kensara et al. 2023 (40) Cross sectional study	soaking paper points in the internal surface	16S rRNA gene sequencing on Illumina MiSeq platform	significantly increased microbial diversity in health		Gram positive bacteria, especially Enterococci were found inside implants with peri implantitis
Jezdic et al. 2023 (41) Cross sectional study	Submucosal plaque	Quantitative Reat Time PCR			Porphyromonas gingivalis is involved in osteolysis and progression of mucositis to periimplantitis
Li et al. 2023 (42) Case control study	Submucosal plaque	16S rRNA gene sequencing using the Illumina MiSeq platform	Rothia		Peptostreptococcus, W 50 53, [Eubacterium] Saphenum group, Rikenellaceae RC 9 gut group, Treponema, Tannerella, Filifactor, Phocaeicola, Desulfobulbus, Fretibacterium, [Eubacterium] nodatum group, Defluviitaleaceae UCG -011, Porphyromonas
Fragkiouda et al. 2024 (43) Cross sectional study	plaque	Quantitative Real Time PCR.			Staphylococcus epidermidis
Almeslet et al. 2024 (44) Cross sectional study	submucosal plaque samples	Culture		Submucosal yeast carriage Smokers	
Hu et al. 2024 (45) Cross sectional study	PICF Cementum	tissue culture plate method and Safranin-O staining			Nicotine promoted growth of Porphyromonas gingivalis, Streptococcus sanguinis and Fusobacterium nucleatum
Park et al. 2024 (46) Case series	Biopsy	PCR and immunohistochemistry			Cutibacterium acnes in cytoplasm of macrophages
Kensara et al. 2024 (47) Clinical Cross sectional study	plaque	16S rRNA gene sequencing on the Illumina MiSeq platform	Gram positive bacteria such as Streptococcus salivarius Prevotella melaninogenica, L wadei and Actinomyces species		Gram negative bacteria like Capnocytophaga leadbetteri, Treponema maltophilum, Peptostreptococcus, Neisseria, Porphyromonas gingivalis, Porphyromonas endodontali, Lactococcus lactis and Filifactor olocis
Dutra et al. 2024 (48) Pilot study	Subgingival biofilm	16S rRNA gene sequencing on the Illumina MiSeq platform	Implants have higher inflammatory background than teeth	Proinflammatory response to biofilm is greater than around teeth	Periimplant microbiome differed significantly from periodontal microbiome
Tocarruncho et al. 2025 (49) Cross sectional study	submucosal microbiome	next generation genomic sequencing Illumina	Anoxibacillus flavithermus, Hemophilus parainfluenzae and Mogibacterium diversum		Filifactor olocis, Porphyromonas endodontalis, Tannerella forsythia, Treponema denticola, Peptostreptococcaceae, [Eubacterium nodatum], Desulfobulbus species HTM 041, Mogibacterium timidum

**Table 4:** Microbial diagnostics after periimplantitis therapy

Study & microbial diagnostic technique	Adjuvant antimicrobial strategy	Primary management of periimplantitis	Outcome
de Waal et al. 2015 (50) Randomized Controlled Trial	Implant surface decontamination with 2% Chlorhexidine (CHX) and 0.12% Chlorhexidine+ 0.05% cetyl pyridinium chloride	Surgical – bone recontouring and apically positioned flap	Use of Chlorhexidine reduces anaerobic bacterial load on implant surface but does not improve overall clinical, radiographic or microbial outcomes
Rakašević et al. 2016 (51) Randomized Controlled Trial	Implant surface decontamination with adjuvant Photodynamic Therapy (PDT) compared to application of chlorhexidine gel	Surgical	PDT caused significant and superior elimination of anaerobic bacteria compared to CHX
Caccianiga et al. 2016 (52) Pilot study Real Time PCR	PDT used as per a High-Level LASER Therapy protocol	Non-Surgical and Surgical therapy	Clinical and radiographic improvements with decreased counts of all bacterial species except E corrodens 6 months after therapy
Hallström et al. 2017 (53) Randomized controlled trial DNA-DNA checkerboard hybridization	Systemic antibiotic therapy using Azithromycin compared to controls without antibiotic therapy	Surgical therapy – open flap debridement	No significant differences between groups
Renvert et al. 2017 (54) Cohort study DNA-DNA checkerboard hybridization	–	Non-surgical management	Bacterial counts for Actinomyces israelii, Aggregatibacter actonomycetemcomitans (Y4), Campylobacter gracilis, Echerichia coli, Fusobacterium periodonticum, Leptotrichia buccalis, Parvimonas micra, Staphylococcus haemolyticus, Streptococcus anginosus, and Tannerella forsythia. Increasing levels of S. Aureus (r2 = 0.856) were found only at implants with non-stable outcome
Stewart et al. 2018 (55) Randomized Controlled Trial DNA-DNA checkerboard hybridization	Toothpaste containing 0.3% triclosan	Anti-infective surgical therapy	Red complex pathogens were only reduced in the test group at 24 months
Galofré et al. 2018 (56) Randomized Controlled Trial	Oral probiotic, Lactobacillus reuteri Prodentis lozenges compared to mechanical therapy alone	Non-surgical mechanical therapy	Greater improvement in clinical parameters but limited effect on peri implant microbial flora
Tada et al. 2018 (57) Randomized Controlled Trial PCR invader method	Lactobacillus reuteri probiotics combined with azithromycin	–	Probiotics prevent inflammation by affecting host responses rather than improving microbial flora in peri-implant sulci
Ramón-Morales et al. 2019 (58) Cross sectional study MALDI-TOFMS	Cemented versus screw retained restorations	–	Gram-negative enteric rods/Pseudomonas and peri implantitis was associated with cemented restored implants.
Shibli et al. 2019 (59) Randomized Controlled Trial DNA-DNA checkerboard hybridization	Adjunctive systemic Amoxicillin and Metronidazole	Non-surgical mechanical debridement	Did not improve clinical and microbiological outcomes
Nie et al. 2020 (60) Case Control study Pyrosequencing	–	Non-surgical mechanical debridement	No significant change in subgingival microbial flora of periimplantitis
Hentenaar et al. 2020 (61) Pilot study Quantitative PCR	Pocket irrigator/evacuator device (IED)	Non-surgical therapy	IED does not resolve periimplantitis or bacterial counts
Wang et al. 2021 (62) Cohort study 16S rRNA gene sequencing	Machine learning-assisted immune profiling into low risk and high-risk group	Surgical therapy	Low-risk group exhibited suppression of keystone pathogen re-colonization. Fusobacterium nucleatum and Prevotella intermedia were significantly enriched in high-risk group
Wang et al. 2021 (63) Cohort study 16S rRNA gene sequencing	Antimicrobial photodynamic therapy	Mechanical debridement	Increased the abundance of beneficial bacteria and decreasing harmful bacteria
Irshad et al. 2021 (64) Retrospective study Culture	Systemic antibiotic Amoxicillin and Metronidazole	Implant surface debridement	Presence and proportions of bacteria did not differ significantly. However, P. Intermedia and P. Micros showed a significant reduction in antibiotic at the recall visit
Sun et al. 2022 (65) Prospective study 16S rRNA gene sequencing		Non-surgical mechanical debridement	Increased complexity of microbial network and significant decrease in pathogenic species
Monje et al. 2022 (66) Retrospective study	Implantoplasty	Surgical therapy	Improved resolution of peri implantitis
Blanco et al. 2022 (67) Randomized Controlled Trial Quantitative PCR	Systemic metronidazole	Non-surgical mechanical debridement	Significantly greater decrease in porphyromonas gingivalis, tannerella forsythia, and campylobacter rectus counts
Di Gianfilippo et al. 2023 (68) Randomized Controlled Trial Quantitative PCR	Er:Yag laser assisted therapy	Surgical therapy	Laser therapy did not alter microbial profile but modulated inflammatory response
Yu et al. 2024 (69) Observational Study 16S rRNA gene sequencing		Non-surgical therapy	No alteration of peri implant microbiome except Rothia aeria
Riben Grundström et al. 2024 (70) Randomized Controlled Trial Quantitative PCR	Systemic antibiotics Amoxicillin+ Metronidazole or Penicillin V+ Metronidazole	Surgical therapy	Improved marginal bone level stability
Siwach et al. 2025 (71) Comparative study Culture	810 nm Diode laser or Photodynamic therapy	Mechanical debridement	Bacterial counts decreased in both groups but no significant difference between groups
Partido et al. 2025 (72) Randomized Controlled Trial 16S rDNA gene sequencing	Glycine powder air abrasive debridement	Ultrasonic debridement	Increased species richness and beneficial microorganisms, loss of pathobionts

**Table 5:** Diagnostic biomarkers of periimplantitis in PICF/Saliva

Study	Biomarker	Sample	Diagnostic technique	Outcome
Severino et al. 2016 (73) Comparative study	IL-6, IL-10, IL-17 and IL-33	PICF Parotid Gland Saliva	ELISA	IL-6, IL-17, IL-33 were elevated in periimplantitis compared to health IL-17, IL-33 increased in peri-implant mucositis compared to health. No difference in saliva
Negri et al. 2016 (74) Cross sectional study	Interferon (INF)-g, interleukin (IL)-4, IL-17, IL-1β, IL-10, IL-6, IL-8, tumor necrosis factor (TNF)-a, matrix metalloproteinase (MMP)-2, MMP-9, osteoprotegerin (OPG), soluble receptor activator of nuclear factor-kb ligand (RANKL), osteocalcin (OC), osteopontin (OPN), transforming growth factor (TGF)-b, and cross-linked telopeptide of type I collagen (ICTP)	PICF	Multiplexed bead immunoassay	Decreased IL-4, TNF-α, and OPG levels and an increased ICTP and TH1/TH2 ratio in peri-implant crevicular fluid in smokers compared to non-smokers
Wang et al. 2016 (75) Cross sectional study	IL -1β and VEGF MMP-8 and Tissue Inhibitor of Matrix Metalloproteinases-2 (TIMP-2) Osteoprotegerin OPG	PICF	Quantibody arrays	IL-1β, TIMP-2, VEGF and OPG increased significantly in peri implantitis MMP 8 was not significantly different
Acharya et al. 2016 (76) Cross sectional	Il-1 β	Saliva PICF	ELISA	Salivary IL-1 β correlated with high PICF IL-1 β score in periimplant mucositis
Janska et al. 2016 (77) Pilot study	MMP-8	PICF	Dentotest aMMP-8	Sensitive method to detect periimplant mucositis and periimplantitis
Che et al. 2017 (78) Comparative study	Lectin- type oxidized LDL receptor – 1 (LOX-1) IL-1β, MMP2 and MMP9	PICF	Western Blot ELISA	LOX-1, IL-1β, MMP2 and MMP9 increased in periimplantitis
Al Ghazal et al. 2017 (79) Randomized Controlled Trial	IL-6, IL-8, IL-1β, TNF, IL-10 and IL-12	PICF	ELISA	Significant correlation between IL-6 and clinical parameter of bleeding on probing
Teixeira et al. 2017 (80) Cross sectional study	Th-17 related cytokines	PICF	Bead based multiplex assay	Mucositis sites in patients having either peri-implantitis, periodontitis or without interproximal alveolar bone loss, expression of Th17-related cytokines in PICF from mucositis sites seem to be similar regardless of the presence or not of alveolar bone loss around implants or teeth
Marques Filho et al. 2018 (81) Case control study	IL-1β, IL–2, IL-4, IL-6, MCP-1, Macrophage Inflammatory Protein (MIP)-1α, MIP-1β, TNF-α	Saliva	Multiplex analysis	No difference between health and periimplantitis
Che et al. 2018 (82) Case control study	Osteopontin (OPN) IL-1β	PICF	Western Blot ELISA	OPN increased in peri implantitis
Alrabiah et al. 2018 (83) Case control study	Advanced Glycation End products (AGEs)	PICF	ELISA	AGEs in PICF were increased in individuals with prediabetes and T2DM. AGES may play an important role in peri-implant inflammation in prediabetes and T2DM
Vohra et al. 2018 (84) Cross sectional Retrospective study	Serum CRP	Serum	ELISA	Serum CRP levels correlated with peri-implant bleeding in obese individuals
Al-Askar et al. 2018 (85) Comparative study	Interleukin-1β and Interleukin-6	Saliva	ELISA	Whole salivary IL-1 β and IL-6 levels were higher among patients with peri-implantitis in non-diabetics. In diabetics parameters are affected by glycemic status
Bhavsar et al. 2019 (86) Case control study	(IL-1β), matrix metalloproteinase-8 (MMP-8), and Macrophage Inflammatory Protein-1α (MIP-1α)	PICF	ELISA	Elevated IL-1β in Periimplantitis Decrease in MMP-8 levels at three months after treatment is consistent with a healing biological response
Algohar & Alqerban 2020 (87) Case control study	Procalcitonin	PICF Saliva	ELISA	Procalcitonin levels in periimplant mucositis and periimplantitis were higher than in health-can be used as surrogate biomarker of periimplant inflammation
Lira-Junior et al. 2020 (88) Comparative study	CSF-1, IL-34, and IL-1β	PICF Saliva	ELISA	Increased levels of CSF-1 in PICF but not in saliva of periimplantitis, can be used to differentiate early from late disease
Alresayes et al. 2021 (89) Comparative study	Cortisol	PICF	ELISA kit (Enzo Cortisol ELISA ADI-900–071)	Volume of PICF collected and levels of cortisol were significantly higher in periimplantitis compared to health
Hentenaar et al. 2021 (90) Comparative study	Pro-inflammatory and anti-inflammatory cytokines IL-1β, TNF-α, IL-6 and G-CSF, collagen degradation enzyme MMP-8, chemokines MCP-1 & MIP-1α/CCL3, bone markers OPG and srankl and interferon-*γ*	PICF	Luminex assay	Significantly increased IL- 1β and MMP-8 levels in periimplantitis No difference in levels of TNF-α, IL-6, MCP-1 and MIP-1α/CCL3, OPG and G-CSF
Djuran et al. 2022 (91) Prospective	RANKL	PICF	ELISA	Baseline levels were significantly increased in peri implant mucositis and peri implantitis, they decreased following treatment and reached healthy controls in peri-implantitis, while in peri implant mucositis RANKL remained significantly increased
Aldulaijan et al. 2022 (92) Comparative study	Alpha Amylase and mucin-4	Saliva	ELISA	Salivary AA levels were significantly high in periimplant mucositis. AA and mucin-4 levels are potential biomarkers for evaluation of peri-implant diseases
Lähteenmäki et al. 2022 (93) Case control study	Active MMP-8 point-of-care (poc)/chairside enzyme-test	PICF	aMMP-8-POC peri-implant sulcular fluid (PISF) lateral-flow immunotests were performed using implantsafe® technology quantitated by oralyzer	The aMMP-8-POC test promptly recorded and reflected peri-implant disease, differentiating it clearly from health. Active and fragmented MMP-8 exhibits a strong and significant association with peri-implantitis as compared to total MMP-8
Teixeira Neves et al. 2022 (94) Prospective study	Strem-1, PGLYRP-1, MMP-8, and TIMP-1	Saliva	ELISA	Significant decrease in strem-1, MMP-8, and TIMP-1 in the peri implantitis group and PGLYRP1 and TIMP-1 in the peri implant mucositis group after treatment
Malmqvist et al. 2024 (95) Cross sectional study	IL-1β, TNF- α, IL-4 and BAFF	PICF	Multiplex immunoassay	IL-1β, TNF-α, IL-4 and BAFF increased in periimplantitis
Alpaslan et al. 2024 (96) Randomized Controlled Trial	Receptor activator of nuclear factor-kappa B ligand (RANKL) and Osteoprotegerin (OPG)	PICF	ELISA	RANKL reductions were significantly higher in the laser group than in the control group OPG levels significantly increased in both
Önder et al. 2024 (97) Prospective cohort study	Calprotectin and MMP-8	PICF	ELISA	Non-surgical treatment of periimplant mucositis and periimplantitis reduced their levels in PICF
Xanthopoulou et al. 2024 (98) Comparative study	aMMP-8 and Azurocidin	PICF	Quantitative point-of-care (POC), chairside lateral flow immunotest ELISA	Significant differences for aMMP-8 levels but not for Azurocidin between health, peri implant mucositis, and peri-Implantitis
Erduran et al. 2024 (99) Randomized Controlled Trial	TWEAK, IL-1β, sclerostin, IL-17, RANKL, OPG and IL-10	PICF	ELISA	IL-17, sclerostin and IL-1β can be used to assess efficacy of periimplantitis treatment
Titusson et al. 2025 (100) Cross sectional study	BAFF, SIL-6RB, IFN-B, SIL-6RA, STNFR-1and Pentraxin -3	Saliva	Multiplex immunoassay panel	Significantly elevated in periimplantitis compared to health

**Table 6:** Metabolic diagnostic techniques in periimplantitis.

Study	Molecule	Technique	Outcome
Doan et al. 2015 (101) Pilot study	536 bp amplicons and 2 kb amplicons in cell free PICF	Conventional PCR	Mucositis group had the highest number, healthy group had low numbers. They can be used as biomarkers to monitor soft tissue inflammation around implants
Sánchez-Siles et al. 2016 (102) Transversal study	Salivary malondialdehyde Salivary myeloperoxidase	High performance liquid chromatography ELISA	These oxidative stress markers are not higher in periimplantitis compared to health
Douillard et al. 2016 (103) Retrospective study	Nerve Growth Factor Expression and Its Receptors trka and p75ntr in periimplant tissue	Immunostaining with antibodies	Intense expression of NGF and trka in the inflammatory cell infiltrate associated with decreased expression of p75ntr in both gingival and pocket epithelium
Mardegan et al. 2017 (104) Case control study	TGF β mRNA, IL-23 mRNA and IL-17 mRNA in gingival tissue	Real Time PCR	IL-23 mrna levels were significantly increased in the peri-implantitis group
Bastos et al. 2018 (105) Case control study	mRNA expression levels for Semaphorins 3A, 3B, 4A, and 4D in per implant tissue	Real Time PCR	Higher gene expression for Sem3A and Sem4D and lower for Sem4A in periimplantitis compared to health
Zhang et al. 2019 (106) Case control study	Receptor Activator of Nuclear factor Kappa-B Ligand (RANKL) in PICF and gingival tissue	ELISA Western blot Immunofluorescence staining	RANKL is involved in peri-implantitis TLR2 and LOX-1 which mediate RANKL production can serve as potential drug targets against peri-implantitis
Yakar et al. 2019 (107) Evaluation study	GCF and PICF levels of Sclerostin TNF-related weak inducer of apoptosis (TWEAK) receptor activator of nuclear factor kappa-beta ligand (RANKL) Osteoprotegerin (OPG)	ELISA	Significantly higher in periimplantitis compared to peri-implant health
Figueiredo et al. 2020 (108) Evaluation study	mRNA of IL-6, IL-1ß, TNF-α, MMP-1, MMP-2, MMP-8, MMP-9, TIMP-1, and TIMP-2 in gingival tissue	Quantitative PCR	IL-1ß mRNA was significantly higher in periimplantitis and IL-6 mRNA was significantly higher in periodontitis and peri-implantitis compared to health. mRNA of metalloproteinases and their inhibitors did not differ between groups
Teixeira et al. 2020 (109) Cross sectional study	Strem-1 and its ligand PGLYRP1 MMP-8 and its inhibitor TIMP-1 in saliva	ELISA	Their levels are increased in inflammation. Strem-1/ PGLYRP1/ MMP-8 axis can be used as biomarkers of peri implant and periodontal diseases
Rakic et al. 2020 (110) Cross sectional	RANKL and OPG	ELISA	Bone turnover markers (BTMs) demonstrated presence of bone resorption in peri implant mucositis; between comparable diagnostic ranges periimplantitis was clinically distinguished from peri implant mucosotis in about 60% of patients while 40% remained diagnosed as false negatives
Jiang et al. 2021 (111) Retrospective study	Serine Protease inhibitors (serpins) family protein expression in PICF	ELISA	Overexpressed in periimplantitis and play an important role in its pathogenesis
Chaparro et al. 2021 (112) Cross sectional study	Extracellular vesicles (evs), and their subpopulations (micro-vesicles and exosomes), and microRNAs (miRNA-21-3p, miRNA-150-5p, and miRNA-26a-5p) in peri-implant crevicular fluid (PICF)	TEM Quantitative Reverse Transcription Polymerase Chain Reaction	An increase concentration of evs with a downregulation expression of mirna-21-3p and mirna-150-5p may be related to development of periimplantitis
Martin et al. 2022 (113) Discovery study	RNA in peri implant tissues	Next-generation transcriptome-wide microarray profiling workflow	Genes involved in actin polymerization, an endosomal-lysosomal pathway were strongly upregulated in periimplantitis
Wang 2022 (114) Observational study	Sirtuin 1 (SIRT1) in PICF	ELISA	Significantly lower in Peri implant mucositis and periimplantitis compared to health
Ahmed et al. 2022 (115) Case control	High mobility Group box chromosomal protein-1 (hmgb-1) tumor necrosis factor-alpha (tnf-α) and interleukin (il)-1β in picf	ELISA	High PICF levels of hgmb 1 is a possible biomarker of periimplantitis
Martins et al. 2022 (116) Case control study	Gene expression of ahr, IL-22, and IL-6 in gingival tissue	RT PCR	Higher gene expression of ahr and IL-6 in periimplantitis. No difference in IL-22 gene expression between health and periimplantitis
Khouly et al. 2022 (117) Pilot study	Global DNA methylation in periimplant gingiva and bone	Global DNA methylation analysis	Higher levels in gingiva compared to bone in implant failure and health reflecting differences in epigenetic response
Ganesan et al. 2022 (118) Pilot study	Transcriptional events at the mucosal-microbial interface in the peri-implant crevice	Illumina hiseq 4000 platform and sequenced using 150 bp paired-end chemistry	Microbial dysbiosis in the peri-implant sulcus promotes a scenario similar to non-healing wound
Chaparro et al. 2022 (119) Pilot study	CCL-20/MIP-3α, BAFF/blys, IL-23, RANKL, and Osteoprotegerin in PICF	ELISA	IL-23 and RANKL may help to elucidate pathogenesis during the conversion from peri-implant health to peri-implantitis
Krishnamoorthy et al. 2023 (120) Pilot study	M6a mRNA METTL3 mRNA METTL3 protein	M6a-RNA methylation quantification kit qRT-PCR Western blot	M6a mRNA, METTL3 mRNA and protein levels were elevated in peri-implantitis
Chen et al. 2023 (121) Evaluation study	Hub genes in periimplant soft tissue	A weighted gene co-expression network analysis validated with qRT-PCR	Hub genes IL10 and IL1B and immune factors CXCL10, IL6, and CXCL12 held highest degree in immune factors network IL 1B can be a therapeutic target
Giro et al. 2023 (122) Case control	Gene expression of IL-4, Macrophage inflammatory protein type 1α (MIP-1α) and MMP-9 in peri implant tissue	Real time PCR	IL-4 gene expression significantly increased in periimplantitis
Djinic Krasavcevic et al. 2023 (123) Observational study	Notch signaling molecules expression levels (Notch1, Notch2, Jagged1, Hes1, and Hey1) Bone remodeling mediators (RANKL and OPG)	Reverse Transcriptase Real Time Polymerase Chain reaction	Notch2 upregulation in RANKL-predominant peri implant mucositis indicates transition to peri implantitis
Halstenbach et al. 2023 (124) Pilot study	PICF proteome analysis	Liquid Chromatography Tandem Mass Spectrometry	Proinflammatory proteins such as immunoglobulins, dysferlin, and S100P, antimicrobial proteins myeloperoxidase, azurocidin were significantly upregulated in periimplantitis
Zhou et al. 2024 (125) Evaluation study	Differential expression analysis of mRNAs, miRNAs, and circRNAs in gingival tissue samples	Illumina hiseq 2500 instrument	Revealed the presence of a heterogeneous circRNA-mediated molecular regulation of periimplantitis. Circrnas are potential diagnostic biomarkers and therapeutic targets
Li et al. 2024 (126) Evaluation study	Single cell RNA sequencing of biopsy	ScRNA-seq	To create a comprehensive single-cell transcriptome profile. There was significantly reduced numbers of stromal cells such as fibroblasts. Immune cells such as monocytes and neutrophils were increased as was differentiation of monocyte/macrophage lineage cells into osteoclasts
Oh et al. 2024 (127) Pilot study	RNA in gingival tissue of periodontitis and periimplantitis	RNA sequencing and bioinformatics analysis	Activated fibroblasts with three marker genes (ACTA2, FAP, and pdgfrβ) overexpressed in peri-implantitis May be used as disease specific biomarkers
Özkan Karasu et al. 2024 (128) Cross sectional study	Oxidative damage biomarkers 8-hydroxydeoxyguanosine (8-ohdg), Malondialdehyde (MDA) and antioxidant enzymes Superoxide dismutase (SOD), Glutathione Peroxidase (gpx) in whole saliva	ELISA Spectrophotometry	Elevated levels of 8-ohdg and MDA indicates onset of peri implant bone loss
Chai et al. 2024 (129) Case control study	Lncrna X-inactive specific transcript (XIST) in saliva	Quantitative Reverse Transcription Polymerase Chain Reaction Bioinformatic prediction Luciferase reporter assay	Expression of XIST positively correlated with periimplantitis
Hamed et al. 2024 (130) Case control study	Expression of miRNA-146a and miRNA-155 in peri implant tissue	Real-time PCR	A significantly higher mean expression of miRNA-155 and miRNA-146a in peri implantitis
Saito et al. 2024 (131) Cross sectional study	Endothelin- 1 (ET-1) in PICF, a peptide derived from vascular endothelial cells	Enzyme immunoassays	Significantly increased expression in peri implant mucositis
Song et al. 2024 (132) Cross sectional study	Metagenomic analysis of peri implant plaque	Metagenomic DNA extraction and Illumina Shotgun Sequencing	Microbiome of periimplantitis is different from health
Chen et al. 2024 (133) Case control study	Biomarkers associated with immune cell infiltration TLR4, CCL3, CXCL8 & IL1βin soft tissue samples	Immunohistochemical staining	Increased expression in peri implantitis
Urvasizoglu et al. 2024 (134) Evaluation study	Molecular markers in saliva CXCL9, CXCL12, CXCL14, mir-4484	ELISA	CXCL14 and mir-4484 differentiates periimplantitis from health and are potential biomarkers of early detection
Soysal et al. 2024 (135) Cross sectional study	Interleukin (IL)-1β, IL-6, IL-10, interferon (IFN)α inflammatory cytokines and the psychological stress-related markers, glucocorticoid receptor-α (grα), and salivary α-amylase (saa) gene expression levels in saliva	Quantitative PCR	Psychological stress may increase the inflammation associated with peri-implantitis by affecting cytokine expression
Oh et al. 2024 (136) Cross sectional study	Differentially expressed genes (DEGs) and related pathways in peri-implantitis	Reverse transcription-quantitative Polymerase chain reaction	CXCL1, CXCL3, MMP9, MMP13, ADAM12, and OSM genes were upregulated in peri-implantitis
Parize et al. 2025 (137) Case control study	Fourier Transfer Infrared Spectroscopy (FTIR) on saliva	FTIR-ATR (Attenuated Total reflectance)	Revealed vibrational nodes of fatty acids, histidine, lipid esters, nucleic acids, tryptophan and is an effective tool for diagnosis
Liu et al. 2025 (138) Cross sectional study	Short Chain Fatty Acids (SCFAs) in saliva	Gas Chromatography Mass Spectrometry & high-performance Liquid Chromatography	SCFAs namely butyric, isovaleric, isobutyric, propionic, acetic, formic and lactic acids correlate significantly and positively with peri implant disease

**Table 7:** Genetics in periimplantitis diagnosis

Study	Gene polymorphism and population	Outcome
Casado et al. 2015 (139) Cross sectional study	BRINP3 genetic variants rs1342913 and rs1935881 Brazilian	Variant rs1342913 and low level of BRINP3 expression are associated with Peri implantitis
García-Delaney et al. 2015 (140) Case control study	IL-1 gene variants IL-1A-C889T, IL-1B+C3953T & IL-1RN+T2018C Caucasian	These variants are not risk factors for PI in heavy smokers
Coelho et al. 2016 (141) Cross sectional study	Polymorphism in BMP4, FGF3, FGF10 and FGFR1 genes individually and in haplotypes	TT polymorphic genotype for BMP4 rs2761884 was associated with healthy peri-implant BMP4 and FGF10 haplotypes are associated with peri-implantitis
Zhou et al. 2016 (142) Case control study	Osteoprotegerin gene (OPG) polymorphisms rs2073617 & rs2073618 Chinese Han	OPG rs2073618 polymorphism is related to risk of PI but not rs2073617
Kadkhodazadeh et al. 2016 (143) Cross sectional study	Natural Resistance associated Macrophage Protein 1 (NRAMP1) gene polymorphism rs2276631 and rs17235409 Iranian	Presence of G allele in both variants is protective against periodontitis but not periimplantitis
Gonsalves et al. 2016 (144) Case control study	Matrix Metalloproteinases 13 (MMP-13), Tissue inhibitor of metalloproteinase type 2 (TIMP-2), Transforming Growth Factor β3 gene variants Brazilian	No association with periodontitis or periimplantitis
Cosyn et al. 2016 (145) Case control study	IL-1α (-889), IL-1β (-511) & IL-1β (+3954) gene polymorphisms Caucasians	IL-1β (+3954) affects osseointegration and causes early implant failure
Petkovic-Curkin et al. 2017 (146) Case control study	Polymorphisms of CD14, TNF-α, IL-6, IL-10, IL-1ra Serbian	smoking and presence of TNFα-308 GA/AA genotypes increased risk for peri-implantitis CD14-159 polymorphic CT/TT genotypes decreased risk
Kun He et al. 2020 (147) Case control study	TNFα-308G/A IL-1α-889C/T IL-1β+3954C/T Chinese	IL-1α-889C/T and IL-1β+3954C/T are associated with risk of Periimplantitis and periodontal status
Chang et al. 2021 (148) Case control study	Epidermal Growth Factor (EGF) gene polymorphism EGF gene rs2237051 EGF gene rs4444903 Chinese Han	GF rs2237051is associated with periimplantitis rs2237051 GG genotype & G allele are protective factors
Saremi et al. 2021 (149) Cross sectional study	Single Nucleotide Polymorphisms (SNPs) of IL-10, IL-1β and TNFα genes Iranian	IL-10-819C/T, IL-10-592C/A & IL-1β+3954C/T increase risk of Periimplantitis No association with TNFα-857G/A and TNFα308G/A polymorphisms
Cardoso et al. 2022 (150) Pilot study	SNPs in IL-1α rs1800587 Il-1β rs1143634 Portugese	Higher frequency of IL-1α gene polymorphism and periimplantitis
Saremi et al. 2024 (151) Case control study	MMP 1,2,3,7 &13 gene polymorphisms Iranian	MMP-3(-11715A/6A) & MMP-7(-181A/G) gene polymorphisms were significantly different between health and periimplantitis
Lafuente-Ibáñez-de-Mendoza et al. 2024 (152) Case control study	single nucleotide polymorphisms (SNP) of inflammatory and bone metabolism related proteins NPs of BMP-4, BRINP3, CD14, FGF-3, FGF-10, GBP-1, IL-1α, IL-1β, IL-10, LTF, OPG and RANKL Basque country	GBP1 rs7911 and BRINP3 rs1935881 were significantly more common in patients with periimplantitis OPG rs2073617 was more frequent in smoker periimplantitis patients and BMP-4 rs17563 or FGF-3 rs1893047 in diabetic periimplantitis patients
Li et al. 2024 (153) Case control study	CD14 gene polymorphisms and peri-implantitis susceptibility Chinese Han	Significant association between 2569190 polymorphisms of CD14 gene GG genotype and G allele were risk factors for PI

**Table 8:** Pathologic diagnostic techniques for periimplantitis.

Study	Tissue	Technique	Outcome
de Araújo et al. 2017 (154) Cross sectional study	Histological changes and immunostaining for CD15, CD57 and HIF-1α in the peri-implant mucosa of patients with and without peri-implantitis	Histology Immunohistochemistry	Increased immunostaining for CD15, a neutrophil marker, and HIF-1α, a tissue hypoxia marker, but no significant difference in immunostaining for CD57, a Natural Killer cell marker in peri-implantitis suggests an active participation of neutrophils and hypoxia in pathogenesis
Kasnak et al. 2018 (155) Cross sectional study	Expression levels of nuclear factor, erythroid 2 like 2 (NFE2L2/NRF2), Parkinsonism associated deglycase (PARK7/DJ-1), kelch-like ECH associated protein 1 (KEAP1), and 8-hydroxy-deoxyguanosine (8-ohdg) in periimplantitis tissue	Immunohistochemistry	Inflammatory cell infiltration in the connective tissue and loss of architecture in the spinous layer of the epithelium in periimplantitis Elevated expressions of 8-ohdg and PARK7/DJ-1
Lucarini et al. 2019 (156) Case control study	VEGF, Microvessel Density, CD34 and CD44 inflammatory biomarkers in interproximal gingival biopsy	Immunohistochemistry	Periimplant Pocket Depth impacts periimplantitis and expression of inflammation markers
Nelson et al. 2020 (157) Cohort study	Micro- and nanosized titanium and ceramic implant-related particles in periimplantitis tissue	Synchrotron *μ*-x-ray fluorescence spectroscopy (XRF), nano-XRF, and μ-x-ray absorption near-edge structure (XANES)	Particle accumulation in inflamed tissues around dental implants
Kulakov et al. 2020 (158) Cross sectional study	Inflammatory and regenerative processes in the patients with developing periimplant mucositis (PM) and peri-implantitis in comparison with the patients with severe periodontitis taking into account the expression of VEGF (vascular endothelial growth factor), SMA (myofibroblastic cell differentiation marker), and Ki-67 (proliferative activity marker)	Histology Immunohistochemistry	Comparing chronic generalized periodontitis and periimplantitis, the latter shows much more pronounced inflammatory and destructive processes around the implant.
Taskan & Gevrek 2020 (159) Case control study	Receptor expressions of peroxisome proliferative-activated receptor (PPAR)-γ, retinoid X receptor (RXR)-α, vitamin D receptor (VDR), and cyclooxygenase (COX)-2 in healthy, periodontitis and periimplantitis tissue	Hematoxylin–Eosin (H & E) and immunohistochemistry	Inflammatory cell infiltration was higher in periodontitis and peri-implantitis while fibroblast cell density had a reverse pattern PPAR-γ and COX-2 expressions were higher in periodontitis and peri-implantitis while RXR-α and VDR was associated with health
Henin et al. 2022 (160) Proof of concept study	Manual and Digital counting of inflammatory biomarkers CD3+, CD4+, CD8+, CD15+, CD20+, CD68+, and CD138+ in periodontitis and periimplant tissue biopsy	Histology and immunohistochemistry	Larger inflammatory infiltrate in Periimplantitis than Periodontitis
Rakic et al. 2022 (161) Case control study	Titanium particles in periimplantitis biopsy specimens and CD68, IL-6, Nf-kb and VEGF markers	Scanning electron microscopy coupled with dispersive x-ray spectrometry Hematoxylin–eosin staining Immunohistochemistry	Free titanium particles interspersed in granulation tissue of periimplantitis And higher proportions of macrophages and intense neovascularization with CD68 and VEGF expression
Ginesin et al. 2023 (162) Cross sectional study	Leukocytes in periimplantitis and periodontitis using gingival biopsy	Flow cytometry	Periimplantitis and periodontitis showed similar proportions of specific (CD4/CD8 ratio of 1.2) and innate (dendritic and NK) immune cells
Villalobos et al. 2024 (163) Case control	Macrophages, neutrophils, NK cells, and blood vessels in peri-implantitis compared to healthy gingiva	Histology and immunohistochemistry	Pro-inflammatory macrophages, vascular architecture and abundant NK cells in peri-implantitis compared to health
Al-Bakri et al. 2024 (164) Pilot study	Neutrophil extracellular traps (nets) in peri-implantitis and periodontitis tissue	Histological, Immunohistochemical (IHC), Immunofluorescence (IF), Transmission Electron microscopy	Higher neutrophil numbers, greater connective tissue destruction and a greater Expression of NET-related markers in periimplantitis
Sahrmann et al. 2024 (165) Case control study	Blood samples were obtained from the basilic vein to assess MA-related laboratory parameters	IgA, macrophage stimulation test on Tio2 (tnf-α and IL-ß) and the analysis of the genetic cytokine profile	Parameters related to Tio2-sensitization of tissue macrophages consistently demonstrated no association with clinical symptoms of peri-implantitis
Rakic et al. 2024 (166) Controlled clinical study	VEGF correlation to titanium particles in biopsy of peri implant granulations	Immunohistochemistry Scanning electron microscopy and dispersive x-ray spectrometry	VEGF reveals neovascularization in periimplantitis Without variation around titanium particles

Types of studies included were Case control (29%), Cross sectional (24%), Controlled clinical trials (9.8%), Pilot studies (9.2%), Case series and case reports (1.8%). Evaluation studies, transversal studies, and proof of concept studies constituted smaller numbers.

## 5 Discussion

This scoping review identified 162 studies and mapped diagnostic techniques used for peri-implant diseases, revealing substantial heterogeneity across study designs, diagnostic criteria, sampling methods, and analytical tools. Most studies use consensus report of the 2017 World Workshop on the Classification of Periodontal and Peri-Implant Diseases and Conditions (3) clinical and radiographic criteria to diagnose periimplantitis and then further assessed novel techniques and protocols for early diagnosis.

### 5.1 Radiographic assessment

Radiographic assessment of peri-implant bone loss is a primary diagnostic approach, employing techniques such as Intraoral Periapical (IOPA) radiographs, panoramic radiographs, Cone Beam Computed Tomography (CBCT), and Computed Tomography (CT). Though IOPA radiographs remain a primary modality to assess peri implant bone level, clinical techniques like bone sounding without flap elevation offer superior accuracy (6). To overcome drawbacks of two-dimensional imaging CBCT is an alternative, but its role in imaging of peri implant bone morphology is limited due to occurrence of metallic streaking artifacts and higher radiation dose. Two-dimensional IOPA radiographs provide good resolution and detail and remain the clinical standard for assessing peri-implant bone loss (7). In our review all studies used intraoral radiographs to assess peri implant bone loss. Intraoral scans have been used to detect volumetric periimplant soft tissue changes. Another novel diagnostic approach evaluated in later studies, was the use of intraoral ultrasonography to assess peri implant soft tissues and crestal bone (8, 12). Their detection of increased peri implant soft tissue volume, as would be seen in peri implant mucositis before occurrence of any bone loss, uniquely positions these diagnostic techniques as potential aids for assessment of peri implant mucositis before occurrence of peri implantitis.

### 5.2 Etiology and therapeutic focus

Etiology of peri-implant inflammation can be multifactorial—including surgical, prosthetic, and plaque-induced triggers as noted by Cannulo et al. in 2016 (167). The implant disease risk assessment (IDRA) tool proposed by Heitz-Mayfield et al. in 2020 included eight risk factors for peri-implantitis namely presence and susceptibility to periodontitis, compliance with periodontal maintenance therapy, clinical, radiographic and prosthodontic factors (168). Research identified in our review predominantly concentrates on plaque-induced disease. These investigations have centered on two main areas, firstly, identifying the specific microbial community structures and dysbiotic “shifts” that initiate the inflammatory cascade, The microbial diagnostic techniques used in many of these studies are Polymerase Chain Reaction (PCR) and Quantitative Real Time Polymerase Chain Reaction (q-PCR). These DNA based techniques can identify microorganisms, but they do not distinguish live from dead bacteria. RNA based methods allow assessment of live bacterial load. Gene sequencing techniques like 16S r RNA gene sequencing allow species level bacterial identification while shotgun metagenomics allows species and strain resolution of multi kingdom microorganisms (bacteria, viruses, fungi and other microorganisms) in a given sample. To name a few organisms, gene sequencing has identified genera Actinomycetes, Rothia and Streptococci with health, Fusobacterium and Prevotella as potential microbial markers of peri implant mucositis and Porphyromonas, Tanerella, Treponema and Fusobacterium with peri implantitis (37, 38). In our review, Real Time PCR was the preferred microbial diagnostic technique in research studies followed by 16S rRNA gene sequencing.

Secondly, investigations have been directed towards evaluation of therapeutic effectiveness of treatments for established peri implant disease. A significant portion of this therapeutic research focused on determining the adjunctive benefit of antimicrobial agents when used with mechanical or surgical debridement to treat periimplantitis. For these studies preferred microbial diagnostic techniques should provide quantitative data of species and their proportions, decrease or elimination of pathogens would thereby indicate therapeutic benefit. Gene sequencing and DNA – DNA checkerboard hybridization were the preferred techniques in majority of studies, quantitative PCR in a smaller number of studies. The DNA-DNA Checkerboard hybridization uses DNA probes, provides quantitative data and is relatively inexpensive.

Very few studies used microbial culture techniques. Two studies used chairside microbial diagnostic test kits namely IAI PADO test (22) and Guidor Perio-implant diagnostic test (56), both of which use quantitative PCR for microbial detection and quantification.

### 5.3 Biomarker and molecular diagnostics

A significant trend in recent research is the pursuit of pre-clinical diagnostics through the analysis of the host inflammatory response, which precedes overt clinical signs. This involves profiling inflammatory biomarkers in peri-implant crevicular fluid or saliva using immunoassays. Enzyme Linked Immunosorbent Assay (ELISA) was the preferred technique in majority of studies reviewed. ELISA can be used for qualitative and quantitative analysis of one biomarker at a time. Multiplex immunoassays measure multiple biomarkers in a single sample simultaneously; however, they require specialized equipment and expertise. Chairside point-of-care tests to detect *active* MMP-8 (not total MMP-8) which exhibits strong correlation with periimplantitis have been used in a few studies (77, 93, 98).

Cytokines- IL-1β, Tumor necrosis Factor (TNF)-α, IL-6, IL-8, Collagen degradation enzymes- Matrix Metalloproteinases (MMPs) are viable candidates showing positive correlation with peri implant inflammation. Furthermore, bone turnover markers (BTMs) like RANK, RANK-ligand (RANKL), osteoprotegerin (OPG), cathepsin-K, and sclerostin provide measurable data on bone metabolism, with the RANKL/OPG axis being a particularly reproducible indicator of real-time bone resorption (110).

In our review, pro-inflammatory cytokine Interleukin-1β (IL-1β) showed a positive correlation with peri implant inflammation in most studies. Peri implant mucositis shows significantly increased levels of IL-1β, aMMP8, procalcitonin, bone turnover marker RANKL and salivary alpha amylase while IL-1β, VEGF, MMP8, Procalcitonin, RANKL, Osteoprotegerin (OPG) are all significantly elevated in peri implantitis. IL-6 exhibits correlation with bleeding on probing (79).

The discovery of novel biomarkers is increasingly driven by “omics” technologies (genomics, transcriptomics, proteomics, and metabolomics), which enable the analysis of comprehensive molecular signatures. Non-targeted metabolomics offers an overview of metabolites within a sample that can be used to correlate disease status and metabolic mechanisms. Targeted metabolomics allows quantification and verification of a specific group of metabolites as per hypothesis. Non targeted metabolomics of PICF in periimplantitis reveals increased levels of polypeptides, amino acids, fatty acids and nucleotides while targeted metabolomics has correlated elevated succinic acid levels with pathogenic microbiota and inflammation (169).

Despite these promising avenues, our review found that no single biomarker has been standardized or universally advocated for the diagnosis of peri-implant mucositis or peri-implantitis.

### 5.4 Genetic predisposition

Research has also been directed at the genetic basis for susceptibility to peri-implant disease, analogous to studies that have linked genetic variants (e.g., in the BRINP3 gene) to periodontitis (170). The focus is on genetic polymorphisms that can alter an individual's immunological response to microbial biofilm. As these polymorphisms are constant and detectable before disease onset, they hold significant potential for early risk assessment and prognosis. The genetic studies included in this review were conducted across diverse populations, including Brazilian, Caucasian, Iranian, and Chinese cohorts.

### 5.5 Pathological and material-related investigations

Pathologic techniques, primarily histology and immunohistochemistry on biopsy specimens, are used to identify changes in tissue architecture and inflammatory markers. A consistent finding is that inflammation-induced destructive processes appear more pronounced around implants compared to natural teeth. Additionally, the role of the implant material itself has been investigated (164). One hypothesis suggested that corrosion of titanium surfaces could release particles into adjacent tissues, triggering persistent inflammation (171). Titanium particles are released into surrounding soft and hard tissues at the time of implant placement, under load and during maintenance therapy (172). These particles potentially trigger inflammation through epigenetic alterations, interfere with signaling pathways and microbial interactions (173). A study by Fretwurst et al. in 2016 found titanium and metal particles in biopsied periimplantitis tissue (174). In our review, studies conducted by Nelson et al. (157) and Rakic et al. (161, 166) have correlated higher concentrations of implant particles (titanium and/or ceramic) with periimplantitis sites. However, there is no evidence to support a causal association between titanium particles and peri-implantitis, suggesting alternative materials like ceramic or zirconia may not offer a significant advantage in this specific regard.

This review is limited by the absence of formal risk-of-bias assessment, consistent with its scoping methodology but restricting evaluation of evidence strength. Only English-language studies published from 2015 to 2025 were included. Considerable variation in diagnostic definitions, imaging parameters, biomarker thresholds, and microbial methods limited the comparability of findings across studies.

## 6 Conclusion

To conclude, this scoping review identifies major diagnostic domains including imaging techniques, biochemical markers, microbial profiling, genetic and histopathologic techniques. Advanced imaging tools, promising biomarkers such as aMMP-8, IL-1β and RANKL and emerging sequencing technologies demonstrate potential utility, although no single standardized diagnostic test currently exists. Future research should prioritize standardized diagnostic criteria, consistent sampling protocols, and high-quality prospective study designs to validate emerging diagnostic approaches.

## Data Availability

The original contributions presented in the study are included in the article/Supplementary Material, further inquiries can be directed to the corresponding author.
